# The association between statistical learning and language development during childhood: A scoping review

**DOI:** 10.1016/j.heliyon.2023.e18693

**Published:** 2023-07-26

**Authors:** Regina Abreu, Svetlana Postarnak, Valentin Vulchanov, Giosuè Baggio, Mila Vulchanova

**Affiliations:** a Language Acquisition and Language Processing Lab, Norwegian University of Science and Technology – Trondheim, Norway; b University of Porto, Portugal

**Keywords:** Statistical learning, Language outcomes, Infants and children, Sensory modality

## Abstract

The statistical account of language acquisition asserts that language is learned through computations on the statistical regularities present in natural languages. This type of account can predict variability in language development measures as arising from individual differences in extracting this statistical information. Given that statistical learning has been attested across different domains and modalities, a central question is which modality is more tightly yoked with language skills. The results of a scoping review, which aimed for the first time at identifying the evidence of the association between statistical learning skills and language outcomes in typically developing infants and children, provide preliminary support for the statistical learning account of language acquisition, mostly in the domain of lexical outcomes, indicating that typically developing infants and children with stronger auditory and audio-visual statistical learning skills perform better on lexical competence tasks. The results also suggest that the relevance of statistical learning skills for language development is dependent on sensory modality.

## 1 Introduction

The exact mechanisms which infants and children rely on in order to acquire the rules of language is still an open and widely debated question [1,2]. Infants and children can identify linguistic units and acquire rules from complex and noisy streams of speech in which word boundaries and grammatical structures are rarely self-evident. They are also able to successfully map word labels to referents that are available in rich and complex visual scenes. A viable question is whether they can extract the relevant information from their linguistic context and, if so, what mechanisms may support this process. Following the *poverty of the stimulus argument*, which suggests that the input to which children are exposed is not rich enough to acquire the target grammars [3], a possibility is that they operate within a constrained search space of possible grammars (e.g., of the type consistent with Universal Grammar), offering innate constraints on possible languages [[4], [5]]. Alternatively, it has been proposed that language acquisition is subserved by the extraction of statistical information from sensory input, i.e., statistical learning (e.g., Ref. [6].

Statistical learning (SL) can be defined as the implicit and rapid process of extracting regularities present in the structure of the sensory input [7]. Naturally produced speech includes many cues through which its structure can be extracted (e.g., transitional probabilities, non-adjacent dependencies, co-occurrence of word-object pairs), and empirical research shows that infants, children and adults are sensitive to these statistical patterns (for a review, see Ref. [8]). [9] suggest that statistical learning is both a broad mechanism, which operates across sensory modalities and domains, but also input specific, by adapting its computations to the nature of the input and the learning context. As such, they argue, it provides a reliable framework for studying and explaining language development, as well as bridging laboratory experimentation with real-life situations, such as multilingualism and variation in developmental trajectories.

Despite the fast growth of the field of SL and the already established tradition of language acquisition research, the evidence of a direct impact of SL skills on language learning outcomes is still limited. While a couple of influential theoretically-motivated reviews published over the last decade [[9], [10], [11]] establish the need for longitudinal research and lay out a framework for future studies, the direct association between SL and language competence across language domains and age-groups has not been systematically reviewed. Furthermore, most studies addressing SL and its association with language competence have focused on adults. Our aim here is to review the evidence of associations between SL in different domains and modalities and language competence, across infancy and childhood.

### 1.1 The role of input modality in statistical learning

SL studies typically involve exposing participants to an artificial language, whose statistical structure mimics the complex structure of natural languages. Crucially, one or more regularities of interest are manipulated, while keeping all other statistics constant (e.g., manipulating transitional probabilities between syllables, while controlling for syllable and word length and acoustic features). Participants are then tested on their implicit and/or explicit learning of the artificial language, using behavioural or neurophysiological methods. Importantly, this paradigm only measures SL ability, but not natural language outcomes or concurrent language knowledge.

SL has been amply documented for auditory speech stimuli. The SL experimental paradigm has shown that word segmentation, phonological learning, and syntactic learning can be performed solely based on statistical cues [8]. From a developmental point of view, studies show that the ability to extract statistical regularities from a speech stream is present from birth [12], and that by twelve months, infants can extract multiple statistics (e.g., transitional probabilities and word-order) present in the input [13]. Importantly, infants treat sequences of artificial speech learned through statistical cues as real-language words: such segments are attended to in a way similar to natural language words [14] and are mapped onto novel objects [15].

Given the nature of SL as a powerful broad cognitive mechanism, an important question is how it manifests in other modalities and to what extent its operation and manifestation in other modalities may be relevant for language. Developmental research has shown that SL is also a crucial mechanism for learning non-auditory and non-linguistic material. Infants' and children's ability to extract statistics from visual [[16], [17], [18]] and non-linguistic auditory input [18,19] is well documented. SL in the visual domain can be expected to be relevant for language acquisition. This can be easily stipulated on the basis of the relevance of visual input in word learning [20], but also from the point of view of mechanisms assumed to support the acquisition of grammar (e.g., the lexical bootstrapping hypothesis, [21,22]; and the semantic bootstrapping hypothesis, [23,24]. Yet, only a handful of studies have examined the developmental trajectory of visual SL [17,18,25] and the possible association between visual SL and language skills is largely understudied. Similarly, although there is evidence that the tactile modality mediates learning in a particular way [26], the trajectory of tactile SL across development has not yet been investigated.

### 1.2 Statistical learning, individual differences and language acquisition

The increasing number of studies showing children's and adults' ability to extract statistics from linguistic material has contributed to the SL account of language acquisition (e.g., Refs. [9,10]). This account is mostly based on the claim that, if humans are able to extract statistical regularities from linguistic input, and if this input provides cues to its structure, then they must acquire language through statistical computations. This is a vulnerable argument, and more consistent approaches to this question have been called upon [8,27]. More importantly, the SL account of language acquisition predicts that at least part of the variability of children's language proficiency is due to individual differences in extracting statistical information. Research on individual differences is therefore crucial, because it is theoretically expected that children with stronger SL skills will perform better on language learning tasks [8,11].

While extant research suggests that SL may indeed be positively associated with language development in both infants and children, there is also evidence of null correlations. It has been suggested that this association might be more complex than what the first theoretical accounts have predicted, because SL is likely to be componential [27]. Different aspects and modalities of SL might tap into different domains of language development at different points in development [7], which demands an analysis of the literature that organizes evidence across modalities and domains. While earlier research and reviews of it have primarily focused on providing evidence of SL and establishing the conditions under which it operates [9], the studies that have specifically tested the association between language skills and SL have not yet been subject to a comprehensive review. Such a review should also include studies that employed statistical regularities from other modalities (e.g., visual regularities distributed in space). If domain-general SL skills predict language outcomes, this could indicate that extraction of statistical information – as a general cognitive mechanism, and independently of the input characteristics – is required for language learning. Thus, the main goal of the current review was to summarize the evidence of impact of SL skills in different modalities on language outcomes in infants and children and to identify gaps in the emerging tradition which bridges between the SL tradition and language acquisition research.

### 1.3 The present study

We conducted a preliminary search for existing scoping and systematic reviews on February 9, 2021 in PROSPERO, Cochrane Database of Systematic Reviews, and Open Science Framework (OSF) database. No reviews were found that examined the association between SL and typical language development in children. Given the absence of such a review in the literature, we performed a scoping review [28] of primary studies that tested the association between SL skills and language proficiency in typically developing infants and children (0–12 years old). We did not perform a meta-analysis of the extracted studies as a result of the lack of homogeneity in study design, stimuli and measures used, as will become clear in the results and discussion below.

#### 1.3.1 Review objectives

This review set out the following objectives:

(a) to identify and assess the body of evidence on the association between SL skills and language proficiency during childhood;
(b) to organize this evidence according to: age (from 0 to 12 years of age); language domain; sensory modality of the SL; characteristics of the SL measure and type of stimuli of the SL task
(c) to summarize the evidence of a statistical account of language acquisition, based on the review of these empirical studies;
(d) to identify gaps in research and domains of evidence.

## 2 Inclusion criteria

### 2.1 Participants

Studies that included and reported results from typically developing children between 0 and 12 years of age were considered. We decided not to include studies and/or results from atypical populations, since the association between language impairment and SL has already been subject to a meta-analysis (see Ref. [29]; and [30]). Regarding the age limit of the children included in studies, the rationale was that, while acquisition of morphology and core grammar in L1 may be complete by 4–5 years of age [31], many relevant skills continue to develop later and into the school years, such as syntax, literacy, figurative language comprehension, and meta-linguistic skills [32,33], L2 acquisition, and sensory processing abilities – i.e., visual SL [25]. Inclusion of studies measuring these skills is pertinent, and therefore participants’ age limit was set to 12 years of age.

### 2.2 Concept

Included studies reported a quantitative measure of language development or language competence and a quantitative measure of SL abilities from the participants and analyzed statistically the association between the two measures. Regarding language measures, tasks or tests of proficiency in the phonology, vocabulary, morphology, syntax, and literacy domains, and overall language (tests which tap into several domains) were considered. Regarding the SL measures, we considered experimental paradigms where participants were familiarized with a natural or artificial language, in any sensory modality (unimodal or multimodal), and then tested on their learning of a given probabilistic rule or pattern embedded in the language, by giving an implicit or explicit response.

### 2.3 Context

The studies were conducted in a controlled laboratory setting in any geographical location.

### 2.4 Types of sources

Sources of information were limited to primary research studies published in peer-reviewed journals and in English. Research on SL typically employs experimental paradigms from which quantitative measures are obtained. We therefore limited our sources to quantitative studies, both with cross-sectional and longitudinal designs. No timeframe restrictions were imposed on the search.

## 3 Methods

This review adopted the Joanna Briggs Institute (JBI) methodology for scoping reviews [28,34]. The objectives, inclusion criteria and methods for this scoping review were specified in advance and documented in a protocol submitted at Open Science Framework (registration https://doi.org/10.17605/OSF.IO/CEQH5).

### 3.1 Search strategy

Scoping reviews involve a comprehensive three-step search strategy. First, we performed an initial limited search in PsycINFO, in order to analyze the keywords contained in the title, abstract and index terms of relevant articles. Using these keywords, we developed the following full search strategy: (“statistical learning” OR “artificial language” OR “artificial grammar”) AND (“language” OR “phonology” OR “vocabulary” OR “lexicon” OR “grammar” OR “syntax” OR “morphology”) AND (“infants” OR “toddlers” OR “children” OR “preschoolers” OR “school children”). The search strategy was implemented in four relevant databases: PsycINFO (Ovid), Web of Science, PubMed, and EBSCOHost (see Appendix A for the individual search strategies). Additionally, we searched for more sources of information in the reference list of all the articles included in the review.

### 3.2 Study selection

The study selection process was performed independently by two reviewers. After each step of the process, the reviewers met to compare and discuss their individual results. Disagreements were solved upon joint discussion. Study selection included the following phases: 1) removal of duplicates, 2) screening on title and abstract, 3) publication in peer-reviewed journals check, 4) full-text examination.

### 3.3 Data extraction

In order to fully accommodate all relevant information, the instrument initially developed for data extraction and presented in the review protocol was revised, as data was extracted. The final instrument included extraction fields for design and sample characteristics, language development measures, SL task measures, and the statistical analysis of the association (see Appendix B). Data extraction was independently conducted by the two reviewers and disagreements were resolved through discussion. When data was missing from the research reports, the authors were contacted.

## 4 Results

### 4.1 Results of the study selection

**Fig. 1:**
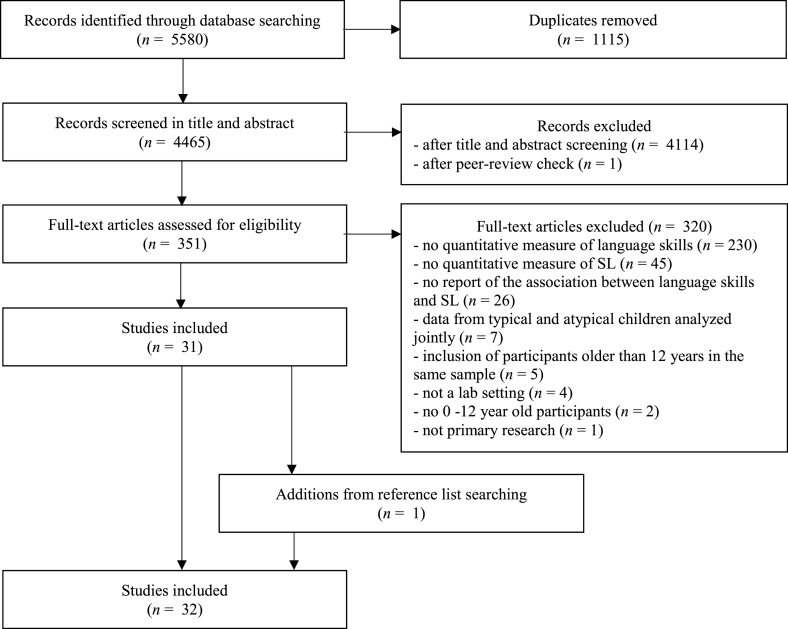
Study selection flow diagram.

The results of the study selection are reported in the flow diagram proposed by the PRISMA Extension for Scoping Reviews protocol (PRISMA-ScR; Tricco et al. [35]), which is consistent with the JBI methodology (see Fig. 1). A total of 5850 sources of information were identified across the four databases. After removing duplicates, 4465 records were left. Title and abstract screening lead to the exclusion of 4114 records. Next, we checked, if the journals where the remaining studies were published, were peer-reviewed, and excluded one record. We then retrieved the full texts of the 351 eligible records. From these, 320 studies were excluded because they did not fulfill one or more criteria for inclusion (reasons stated in the flow chart – Fig. 1). At a final step, we searched in the reference list of the included studies for additional sources of information and included one more record. Thirty-two studies were therefore retained for review.

#### 4.1.1 Design and sample characteristics

Table 1 reports a summary of the characteristics of the included studies. The studies were published between 2001 and 2021, and the sample size ranged between 15 and 553 participants, with a median of 35 participants. The participants age varied from 4 months to 12 years, and their native language was English in 75% of the studies. Only five of the studies had a longitudinal design.

**Table 1:** Descriptive data of the included studies.

		Design & sample				Language development test		Statistical learning task					
No.	Reference	Research design	Age of participants	Native language of participants	Sample size	Language domain	Test name	Perceptual modality	Test name	Stimuli	Statistical pattern	Measure of learning	Obtained Correlations
1	[17]	cross-sectional	6–12 years	English	38	literacy	WRAT	visual	triplet segmentation task	cartoon figures	transitional probabilities	forced choice	control age: r = .327, p < .05
													control grade: r = .364, p < .05
2	[36]	longitudinal	measure 1: 7 months, measure 2: 24 months	Italian	30	lexicon	MCDI, mean length of utterance	visual	artificial grammar learning	shapes	ABB-like rule	eye movement	SL explains 15% of grammar variance
						syntax							
						lexicon							SL predicts grammar: Beta = 0.373; p = .036
						syntax							
													SL does not predict voc: Beta = 0.157; p = .379
3	[37]	cross-sectional	7–11 years	English	20	lexicon	sentence comprehension task, word-picture matching task, TOWRE	visual	serial reaction time task	cartoon figures	transitional probabilities	reaction times	grammar: r = −.48, p < .05
						syntax							voc: r = .20, p = ns
						literacy							reading: words: r = −.24, p = ns, nonwords: r = −.37, p = ns
4	[38]	cross-sectional	8–12 years	English	26	lexicon syntax	PPVT, CASL	visual	oddball task	cartoon figures	transitional probabilities	event-related potentials	ERPs-voc: r (26) = −.14, p = ns
												reaction times	RTs-voc: r (24) = .22, p = ns
													ERPs-grammar: r (26) = −.26, p = ns
5	[39]	cross-sectional	5–12 years	English	78	lexicon	PPVT, EVT	auditory	triplet segmentation task	syllables	transitional probabilities	forced choice	rec. voc: r = .23, p < .05
													exp. voc: r = .28, p < .001
6	[40]	longitudinal	measure 1: 17 months, measure 2: 19–30 months	English	71	lexicon	MCDI	auditory	triplet segmentation task	syllables	non-adjacent dependencies	eye movement	SL & concurrent lang. dev. seg.-exp. voc. size: r = .32, n = 68, p = .008
													seg.-rec. voc. size: r = .32, n = 68, p = .007
													gen.-expr. voc. size: r = −.04, n = 59, p = .762
													gen.-rec. voc. size: r = −.09, n = 59, p = .508
7	[41]	cross-sectional	6–12 years	French	15	syntax	TROG, PPVT	visual	serial reaction time task	cartoon figures	transitional probabilities	reaction times	ECOSSE: touchscreen: r = .24, p = .37,
						lexicon							keyboard: r = .28, p = .31
													EVIP: touchscreen: r = −.075, p = <.05 (indicating positive association between the measures, see notes), keyboard: r = .34, p = .20
8	[42]	cross-sectional	7–11 years	French	21	syntax	TROG, PPVT, ELO	visual	serial reaction time task	cartoon figures	transitional probabilities	reaction times	ELO: r = .11, n = 21, p = .63
						lexicon							ECOSSE: r = .25, n = 21, p = .26
													EVIP: r = .27, n = 21, p = .22
9	[43]	cross-sectional	8–12 years	English	26	lexicon	PPVT, CELF	auditory	triplet segmentation task	syllables	transitional probabilities	forced choice	voc: r = −.072, p = ns
						overall language							lang: r = −.172, p = ns
10	[44]	cross-sectional	6–9 years	English	27	lexicon	PPVT	auditory	artificial grammar learning	words	rule	forced choice	beta = −.004, p = .10
													but interaction with item type: beta = .008, p = .02 (children with higher voc. showed larger distinction between familiar and ungrammatical items)
11	[45]	longitudinal	measure 1: 4 months, measure 2: 8–9 months	French	33	overall language	babbling questionnaire	auditory	triplet segmentation task	syllables	transitional probabilities	eye movement	SL - concurrent babbling: r = −.42, p = .02
													SL - babbling at 4 mo: r = −.08, p = .66
12	[46]	cross-sectional	8–11 years	English	22	overall language	CELF, CTOPP	auditory	triplet segmentation task	syllables	transitional probabilities	forced choice	sentence recall: r = .19, p .39
						phonology							nonword repetition: r = .08, p = .72
													digit recall: r = −.31, p = .16
13	[2]	cross-sectional	4–6 years	English	100	syntax	syntactic priming task, BPVS	visual	serial reaction time task	cartoon figures	transitional probabilities	reaction times	voc: r = .183, p = ns
						lexicon							
14	[47]	cross-sectional	6–8 years	English	68	syntax	language comprehension task, PPVT	visual	triplet segmentation task	cartoon figures	transitional probabilities	forced choice	SL and voc.: r = .22, p = ns
						lexicon							
15	[48]	cross-sectional	7–10 years	Dutch	36	literacy	word reading test, pseudoword reading test, spelling test	visual	triplet segmentation task	cartoon figures	transitional probabilities	forced choice	read.words: r = .08, p = .64
													read.pseud: r = .04, p = .83
													spelling: r = .10, p = .56
16	[49]	cross-sectional	7–10 years	Dutch	35	overall language	CELF	visual	serial reaction time task	cartoon figures	transitional probabilities	reaction times	r = .18, p = .30
17	[50]	cross-sectional	15–16 months	English	38	lexicon	word recognition task, MCDI	auditory	triplet segmentation task	syllables	transitional probabilities	eye movement	voc. size: r = .16, p = ns
													LPE: r = −.38, p < .05
18	[51]	longitudinal	measure 1: 12 months; measure 2: 15 months; measure 3: 18 months	English	34	lexicon	MCDI	auditory	artificial grammar learning	words	non-adjacent dependencies	eye movement	females:
													- rec.voc 12mo & NAD 15mo: r = 0.57, p = .022, n = 16
													- rec.voc 12mo & NAD 18mo: r = −0.65, p = .009, n = 15
													- exp.voc 12mo & NAD 15mo: r = .26, p = ns, n = 16
													- exp.voc 12mo & NAD 18mo: r = −.44, p = ns, n = 15
													males:
													- rec.voc 12mo & NAD 15mo: r = −.20, p = ns, n = 18
													- rec.voc 12mo & NAD 18mo: r = .06, p = ns, n = 15
													- exp.voc 12mo & NAD 15mo: r = −.23, p = .022, n = 18
													- exp.voc 12mo & NAD 18mo: r = .38, p = .009, n = 15
19	[52]	cross-sectional	8–12 years	English	20	lexicon	gating task, word definition task	auditory	triplet segmentation task	syllables	transitional probabilities	forced choice	lex-phon.: r = −.28, p = ns
													lex.sem.: r = −.10, p = ns
20	[53]	cross-sectional	8–9 years	Spanish	26	literacy	LEE, PROESC	visual	artificial language learning	words	graphotactic rule	forced choice	word reading: r = .14, p = ns
													pseudoword reading: r = −.12, p = ns
													word writing: r = .44, p = .023
													pseudoword writing: r = .08, p = ns
21	[54]	cross-sectional	6–9 years	English	31	literacy	WJ, TOWRE, CTOPP	visual	artificial grammar learning	shapes	rule, rule	forced choice	visual SL
						phonolog		auditory		pure tones			- phon.awar.: r = .52, p < .01
													- basic reading: r = .11, p = .81
													- reading fluency: r = .16, p = .81 auditory SL
													- phon.awar.: r = .16, p = .53
													- basic reading: r = .31, p = .27
													- reading fluency: r = .22, p = .53
22	[55]	cross-sectional	30 months	English	36	lexicon	MCDI	audiovisual	cross-situational word learning	actions & verbs	co-ocurrence of word-object pairs	eye movement	r = .22, p = .19
23	[56]	longitudinal	measure 1: 8–9 months; measure 2: 13–14 months;	English	56	lexicon	MCDI	visual	sequence learning task	shapes	transitional probabilities	eye movement	concurrent MCDI (n = 56)
													- voc.comp.: r = .28, p = .04
													- phrases: r = .07, p = .62
													- gesture comp.: r = .17, p = .22
													predictive MCDI (n = 40)
													- voc.comp.: r = .24, p = .15
													- phrases: r = .11, p = .50
													- voc.prod.: r = r = .01, p = .96
													- gesture comp.: r = .34, p = .04
24	[57]	cross-sectional	20 months	English	37	lexicon	MCDI	audiovisual	triplet segmentation task	words & pictures	transitional probabilities	eye movement	high-TP: beta = −.492, p = .001
									ostensive word learning task				low-TP: beta = .276, p = .090
													inf.small voc.: learn high-TP words (t (17) = 2.53, p = .02, d = .60), but not low-TP words (t (17) = −.54, p = .6)
25	[58]	cross-sectional	12 & 14 months	English	48	lexicon	MCDI	audiovisual	cross-situational word learning	words & shapes	co-ocurrence of word-object pairs	eye movement	2 (Age) by 2 (learner/nonlearner) X 2 (Receptive/Productive Vocabulary) anova:
													- main effect of learner/nonlearner: F (1,44) = 13.47, p < .001
													- infants who learned the pairs, were the ones with more advanced vocabularies for their age
26	[59]	cross-sectional	4–10 years	English	553	lexicon	TOLD, OWPVT, CTOPP	auditory	triplet segmentation task Simon task	syllables	transitional probabilities, rule	forced choice, sequence replication	word segmentation: CTOPP-BNW, r = −.10, p < .05; CTOPP-NWR, r = .10, p < .05; EOWPVT = .08, p < .05; and ROWPVT, r = .13, p < .01
						morphology		visual		colour squares			sequence learning (Simon Task): CTOPP-E, r = .09, p < .05; CTOPP-BNW r = .09, p < .05; and CTOPP = MFD, r = .09, p < .05
						syntax							
27	[60]	cross-sectional	3–12 years	English	60	morphology	CELF	auditory	artificial grammar learning	A(B)C sentences	dependency rules	forced choice	r = .387, p < .001
28	[61]	cross-sectional	3–6 years	English	34	phonology	GFTA, PPVT	audiovisual	ostensive word-learning task	words & cartoon figures	phonotactic probabilities	forced choice	vocabulary:
						lexicon							- referent identification: r = .36, p < .05
													- word identification: r = .12, p > .5
													- picture naming: r = −.04, p > .5
													phonology:
													- referent identification: r = −.26, p > .10
													- word identification: r = −.05, p > .5
													- picture naming: r = −.09, p > .5
29	[62]	cross-sectional	7–12 years	Norwegian	65	literacy	TOWRE	visual	triplet segmentation task	cartoon figures	transitional probabilities	forced choice	vocabulary:
30	[63]	cross-sectional	2–7 years	English	19	lexicon	PPVT, familiar word processing tasks	audiovisual	cross-situational word learning	words & cartoon figures	co-ocurrence of word-object pairs	eye movement	receptive vocabulary: r = .37, p = .10
													word processing: r = .46, p = .03
31	[64]	cross-sectional	15-16 & 19–20 months	English	32	lexicon	MCDI	audiovisual	cross-situational word learning	words & objects	co-ocurrence of word-object pairs	eye movement	r = .18, p = .29
32	[65]	cross-sectional	2–5 years	English	47	lexicon	PPVT	audiovisual	cross-situational word learning	words & objects	co-ocurrence of word-object pairs	forced choice	r = .57, p < .05

**Abbreviations:** BPVS: British Picture Vocabulary Scale; CASL: Comprehensive Assessment of Spoken Language; CELF: Clinical Evaluation of Language Fundamentals; CTOPP: Comprehensive Test of Phonological Processing; ELO: Evaluation du Langage Oral; EVT: Expressive Vocabulary Test; GFTA: Goldman-Fristoe Test of Articulation; LEE: Test de Lectura y Escritura en Español; MCDI: MacArthur-Bates Communicative Development Inventories; OWPVT: One-Word Picture Vocabulary Test; PPVT: Peabody Picture Vocabulary Task; PROESC: Evaluacion de los Processos de Escritura; TOWRE: Test of Word Reading Efficiency; TROG: Test for Reception of Grammar; Woodcock–Johnson Test of Achievement; WRAT: Wide Range Achievement Test.

### 4.2 Language development measures

Fifty nine percent of the studies obtained measures in only one language domain, typically the lexical domain. Table 2 shows the frequency of the language domains tested. Most of the language tasks employed were standardized tests, but questionnaires and experimental tasks were also used.

**Table 2:** Frequency of studies that measured each language domain.

Language domain	Percentage (*n*)
Lexicon	72% (23)
Syntax	25% (8)
Literacy	19% (6)
Phonology	13% (4)
Overall language	13% (4)
Morphology	6% (2)

NOTE: Some studies obtained measures from more than one domain, therefore the total percentage in this table is higher than 100%.

### 4.3 Statistical learning measures

Among the 32 studies, there was a total of 34 SL tasks: 13 studies included a visual task, 10, an auditory task, 7, an audio-visual task, and 2 studies had one auditory task and one visual task. The triplet segmentation task, which involves CV syllables organized into triplets and tests whether participants can distinguish between syllable triplets forming words vs. syllable triplets spanning word boundaries, was employed in 67% of the studies measuring SL in the auditory modality, followed by artificial grammar learning tasks (33%). 92% of these studies presented linguistic material, syllables in most cases. In the visual modality, 33% of the studies used the serial reaction time, 27% used the triplet segmentation task, and 20% used artificial grammar learning tasks. Most of the visual SL tasks presented cartoon figures as stimuli. In the audio-visual modality, 5 out of 7 studies used the cross-situational word learning task, with words used as auditory stimuli and, typically, objects as visual stimuli.

The statistical pattern to be learned in SL tasks was typically transitional probabilities (56%), but also a rule (21%), or the co-occurrence of word-object pairs (15%). Half of the studies used implicit measures of learning, the most common ones being eye movements (34% of all studies) and reaction times (19% of all studies), which are online measures. The other half of the studies used explicit measures of learning, particularly a forced choice measure (47% of all studies), which is an offline measure.

### 4.4 SL and language development

**Fig. 2:**
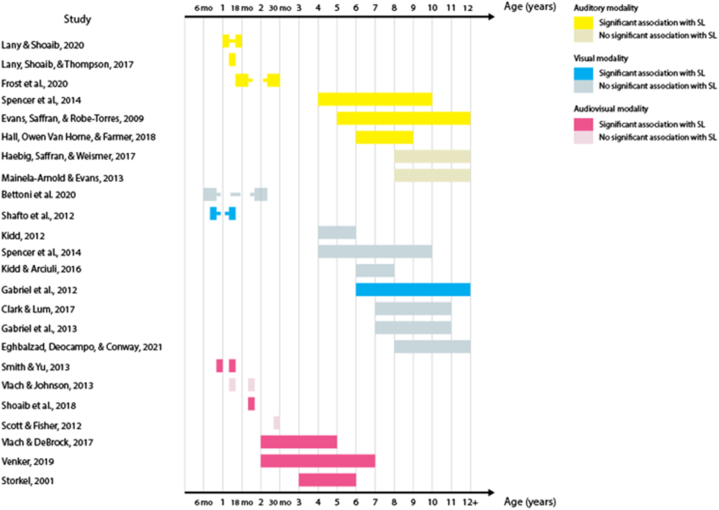
Significant and non-significant tests of association for lexical skills according to age ranges studied.

**Fig. 3:**
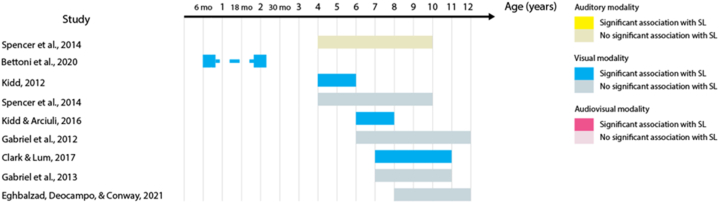
Significant and non-significant tests of association for syntactic skills according to age ranges studied.

**Fig. 4:**
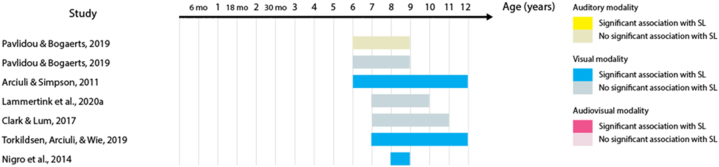
Significant and non-significant tests of association for literacy skills according to age ranges studied.

**Fig. 5:**
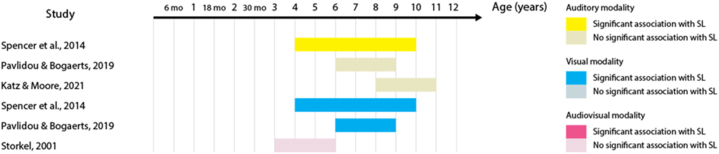
Significant and non-significant tests of association for phonological skills according to age ranges studied.

**Fig. 6:**
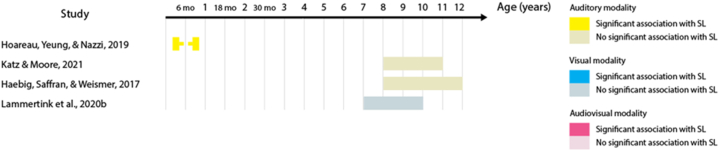
Significant and non-significant tests of association for overall language skills according to age ranges studied.

**Fig. 7:**
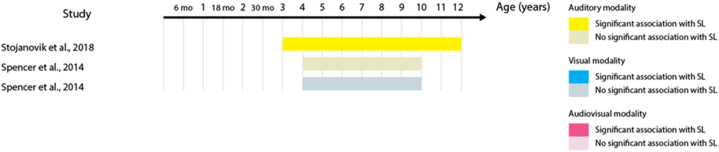
Significant and non-significant tests of association for morphological skills according to age ranges studied.

23 out of the 32 studies (68%) report at least one statistically significant association between the two measures. Across these studies, 53 tests of association were performed – including different sensory modalities and different language domains. When we analyzed these 53 tests of association, the proportion of significant results was reduced to 41% of the tests. Table 3 shows the percentage of significant tests for each language domain. This percentage is largest in the lexical domain. When the proportion of significant tests is analyzed according to SL sensory modality, audio-visual SL has the largest proportion (63% of the eight studies), followed by auditory SL (50% of the 18 studies), and by visual SL (41% of the 27 studies). Fig. 2, Fig. 3, Fig. 4, Fig. 5, Fig. 6, Fig. 7 present diagrams of the results according to language domain, SL modality, age and statistical significance of the association between SL test and language skill tested.

**Table 3:** Frequency of tests of association between SL and each of the language domain.

Language domain	Number of tests	Percentage of significant tests
Lexicon	24	54%
Syntax	9	44%
Literacy	7	43%
Phonology	6	50%
Overall language	4	25%
Morphology	3	33%

### 4.5 SL and lexical development

For infants, auditory SL at 15–16 months correlates with concurrent lexical-processing efficiency, but not with the number of words understood [50]. Furthermore, auditory SL at 17 months correlates with concurrent lexical development and predicts receptive vocabulary up to 25 months and expressive vocabulary up to 30 months [40]. Associations in the opposite direction are also attested. Thus, receptive and expressive vocabulary at 12 months of age is associated with auditory SL skills at 15 and 18 months [51]. Similarly, between 4 and 12 years of age, receptive and expressive vocabulary is associated with auditory SL skills [39,59]. Particularly, existing receptive vocabulary seems to predict the ability to distinguish familiar from unfamiliar auditory items learnt through statistics [44]. However, other studies have failed to find a correlation between auditory SL and receptive vocabulary [43], lexical-phonological access, and lexical-semantic ability [52].

The positive relationship between vocabulary and SL skills is also attested in the audio-visual modality. Thus, 12- and 14-month-olds who learn novel word-object pairs in audio-visual SL tasks are the ones with more advanced vocabularies for their age [58]. However, the vocabulary advantage was not confirmed in older infants, where, at 20 months of age, infants with larger expressive vocabulary sizes were more resistant to learning new words in audio-visual SL tasks [57]. Other studies did not find an association between expressive vocabulary and audio-visual SL in 15-, 20-, and 30-month-olds [55,64]. Results for children between two and seven years of age are also mixed. Two studies found receptive vocabulary to correlate with audio-visual SL [61,65], while another study reports an association with familiar word processing measures, but not with receptive vocabulary [63].

Visual SL skills at 8–9 months of age predict concurrent vocabulary comprehension and gesture comprehension at 13–14 months [58], but not concurrent or later (13–14 and 24 months) expressive vocabulary [36,58]. Most studies have failed to find an association between visual SL skills and receptive vocabulary, expressive vocabulary, and lexical processing speed in 4- to 12-year-olds [2,37,38,42,47,59]. Yet, one study found visual SL to be associated with receptive vocabulary in school-age children [41]. Results are visualized in Fig. 2 above.

### 4.6 SL and syntactic development

The majority of studies which have investigated associations between SL and syntactic development have addressed the visual modality. Visual SL abilities at 7 months of age predict the mean length of utterance at 24 months [36]. In preschool age, visual SL predicts the ability to learn passive forms in a syntactic priming task [2]. In school-age children, visual SL predicts knowledge of low-frequency syntactic structures [47] and correlates with grammatical processing speed [37]. However, other studies did not find evidence of an association with receptive and expressive syntax in 6-to 12-year-olds [38,41,42]. One single study measured both visual and auditory SL skills during childhood, suggesting no statistically significant association of either modality with the ability to understand sentences (Spencer et al., 2014). Results are visualized in Fig. 3 above.

### 4.7 SL and literacy development

Some studies suggest that visual SL skills are associated with reading, writing, and spelling abilities in school-age children [17,53,62], Fig.,4), while others have failed to find the same association [37,48,54]; Fig. 4). The one study that tested auditory SL did not find an association with reading abilities during childhood [54]; Fig. 4).

### 4.8 SL and phonological development

One study found phonological processing abilities to be associated with both auditory and visual SL skills in 4- to 10-year-olds [59]; Fig. 5). Other studies suggest that these abilities might only correlate with visual SL, and not with auditory SL [46,54]; Fig. 5). Audio-visual SL was also not associated with productive phonology in children between 3 and 6 years of age [61]; Fig. 5).

### 4.9 SL and overall language

Auditory SL skills at 8–9 months of age are associated with concurrent babbling ability, but not with babbling ability at 4 months of age [45]; Fig. 6). Other overall language measures were not found to correlate with SL skills during childhood: a composite of lexical and syntactic skills did not correlate with auditory SL [43]; Fig. 6) and sentence recall abilities did not correlate with auditory and visual SL [46,49]; Fig. 6).

### 4.10 SL and morphology

Acquisition of word structure was found to be associated with auditory SL in children between 3 and 12 years of age [60]; Fig. 7). Yet, another study failed to find a statistically significant correlation between morphological comprehension and auditory and visual SL in 4- to 10-year-olds (Spencer et al., 2014, Fig. 7).

## 5 Discussion

Statistical learning has been implicated as a viable candidate to explain the fast rate of language development, given the complexity and richness of the input infants and children are exposed to throughout development [9,10]. It has also been attested in a number of sensory modalities and domains. Thus, the main aim of the current review was to assess the evidence of an association between SL skills and language competence in childhood, thus bridging between research which documents the presence of SL skills independently and research which actually documents its association with language competence across development. An additional goal was to identify the role of SL in different modalities and its impact on language. This review identified 32 studies which measured the statistical association between a proxy of language development and a proxy of SL. Studies cover the whole spectrum of infancy and childhood, however with an uneven distribution, with many studies focusing on young infants or school-age children, and fewer addressing pre-school aged children. Regrettably, most studies do not provide specific reasons for having selected a narrow or a wide age range. The selection of the age range is an important parameter to consider, given that SL preferences and reliance on cues might change with age and over development, as indicated in the review by Saffran and Kirkham [9]. An important open question related to age is the (in)variability of SL and whether and how this applies to different modalities [11,18]. Given the evidence that auditory SL improves in infancy, while visual SL does not change [66] and that the opposite trend has been observed in school-age children [25], it may be speculated that SL mechanisms operate at different time scales and in different directions in the two modalities involved across early development. This may respectively suggest differential impact of auditory and visual SL on language outcomes across childhood. Thus, further studies, including several modalities and using longitudinal designs, are needed to address this question. Furthermore, sample sizes with wide age ranges can be problematic, since SL tasks are not standardized for age. Therefore, the same SL task might not be ideal for children in different stages of cognitive development.

The observable trend for the majority of studies in this review was a cross-sectional design, English-speaking children as participants, and the inclusion of measures of lexical development and either auditory or visual SL skills. Triplet segmentation tasks and artificial grammar learning tasks were the most commonly used paradigms to measure SL skills. Both implicit and explicit measures of learning were used, typically eye movements (online) or forced choice measures (offline).

Since our main aim was to analyze the association between language development and SL skills across language domains and SL modalities, we were looking for evidence of links in different domains of language competence. A large part of the included studies obtained measures of lexical skills, which allows us to draw some relatively stable conclusions on the association between vocabulary development and SL skills. However, the number of studies measuring other language domains might not be sufficient for the same type of conclusions.

Receptive and expressive vocabulary seem to be associated with auditory and audio-visual SL, but not with visual SL alone. This is not surprising, given the central role of the auditory modality in oral language skills and, specifically, in word segmentation. The role of skills in audio-visual binding is also quite consistent with the process of mapping auditory information (phonological form) to visually available referents corresponding to word meanings, which is an essential step in word learning [20]. The reviewed studies attest not only concurrent or predictive relationship between SL and vocabulary, but also attest an association in the opposite direction, namely between existing vocabulary knowledge and SL skills. This finding is consistent with the evidence of a dynamic bi-directional relationship between the ability to acquire language and acquired language competence, as well as (neuro-)constructivist accounts of development [67,68].

The results concerning the visual modality and syntactic skills, literacy and phonological skills are mixed, with some studies suggesting that these language domains are not associated with visual SL, while others have found positive associations. These results may indicate that language development in general might not be directly associated with visual SL. Alternatively, the data may suggest that visual SL may be operative selectively at different stages of language development and for different types of skills. The latter conclusion is particularly plausible, given the wide age ranges studied and the inconsistency of paradigms used. Interestingly, nearly all of the studies which addressed grammar/syntax skills, used a visual SL task and half of those studies found positive associations between a measure of grammar and SL in the visual domain. In addition, the only study which tested auditory SL did not document an association with grammar. This may suggest that statistical learning skills in the visual domain may be more relevant for grammar/syntax, but not for vocabulary learning. However, whether the association found between lexical skills and auditory and audio-visual SL also holds across other language development domains, is still an open question.

From all the tests of the association between language outcomes and SL skills, only 41% were statistically significant. When this percentage is further analyzed according to categories, the largest shares are found for the lexical domain and for audio-visual SL. There is also a tendency for a higher percentage of significant results in language domains that have been studied in more tests (see Table 3). This suggests that the association with SL skills might not be specific to the lexical domain, but simply more evident because of the bias towards that domain. It can be speculated that the strong (er) association between SL skills and word learning may arise from the nature of the tasks used. Given that the majority of studies employed a triplet segmentation SL task which taps the ability to track transitional probabilities and, as such, exactly the skills underlying word learning and segmentation, the current findings are not surprising. Furthermore, it has been shown that SL skills are highly flexible and can adjust depending on the cues available in the input [9]. This suggests that the nature of the SL task in the study design and the manipulations it includes are of paramount importance for the evidence it may provide [17].

The current findings suggest that the role of SL in language acquisition is dependent on the sensory modality of the statistics to be learned. This is based on two results. Firstly, auditory and audio-visual SL are positively associated with, and seem to predict, lexical competence, in at least a proportion of the reviewed studies. Secondly, visual SL is not associated with lexical competence, but may be associated with syntactic and literacy skills, pending further evidence. The primary role of the auditory modality for language development is consistent with findings concerning the differences in the developmental trajectories of SL in different modalities. Thus [66], found evidence of weaker learning in the visual modality than the auditory modality for infants in the age range 8–10 months. This study also documents different developmental trajectories across modalities: while auditory SL increased, visual SL did not change for this age range. Modality-based differences in the development of SL abilities were also documented in the study by Raviv & Arnon [25] of older children, however, in the opposite direction. While children's learning in the visual domain improved with age (between 5 and 12 years), learning in the auditory domain did not change in the tested age range. These findings indicate that SL develops at different time-scales across modalities, and may explain the lack of positive associations in some of the studies reported here, as a result of the age-range of the sample. In addition, the longitudinal study reported in [18] suggests that stimulus type may play a role in measuring the development of SL skills across modalities. They found that both auditory and visual SL develop in childhood (between 5 and 12 years), while SL only for language stimuli remained constant in that age-group, suggesting a primary role of auditory SL for speech in early infant development. A recent meta-analysis of SL research [69] reveals that auditory linguistic SL displays the kind of robustness for it to play a central role in language learning, but more evidence is required. This is also consistent with the evidence from early speech perception research [70].

It has been noted elsewhere that SL tasks lack important psychometric characteristics, and therefore might not be used as a reliable measure of individual differences [11,71,72]. This would explain the variability of some correlation findings between SL skills and language development: the syntactic domain is paradigmatic, with half studies showing a significant association and the other half failing to find significant effects. This indicates that we cannot at present be confident in the degrees and directions of the correlations obtained, and that uncertainty remains, not only at the level of individual results, but also concerning the validity of SL constructs, in particular of stimulus and tasks parameters, as well as of the hypotheses connecting SL to language acquisition. On the one hand, this implies that some of the published results may be spurious or non-replicable, and on the other, that even replicable results may be difficult to interpret in light of plausible models linking SL skills, individual differences, and language acquisition in infancy and childhood.

One of the goals of this review was to identify gaps in research. We found that, while the lexicon has been addressed in the majority of studies included in the current review, all language domains require systematic future research, especially given the statistically inconclusive evidence so far. This would provide a more complete overview on the importance of SL in language acquisition. If auditory SL skills are associated with other linguistic domains, this would suggest that auditory SL skills are basic cognitive abilities for language development in general. However, given the evidence of associations between visual SL and grammar identified in the current review, the possibility arises that other SL skills are selectively associated, and more tightly yoked, with domains, such as grammar/syntax. However, this requires future studies measuring these skills within a direct comparison between auditory and visual SL skills. Other domains in need of detailed future investigation are phonological and morphological skills, since evidence for these domains is scarce.

A serious limitation for interpreting the findings from the reviewed studies is the heterogeneity of the tasks used to measure SL, as well as the language constructs which have been used as dependent variables. For instance, the studies looking at associations between SL and lexical skills and outcomes have selectively studied either overall vocabulary or only receptive or expressive vocabulary or lexical processing, as well as word and gesture comprehension. This reflects inconsistency regarding the hypotheses and the constructs under study.

Studies which include SL tasks across different sensory modalities can provide useful evidence, by allowing a direct, within-subjects comparison across modalities. SL cross-modal comparisons are relevant, since there seems to be an advantage for auditory learning relative to tactile and visual learning [26]. The audio-visual modality is of specific interest for language, given the cross-modal nature of language experience, best observed in word learning where auditory input is mapped onto visual referent candidates [73]. Furthermore, the studies exploring the audio-visual modality are the ones where as many as 63% report significant associations between SL and language outcomes. We did not find studies measuring the association between language development and SL skills in other sensory modalities besides auditory, visual, and audio-visual. Specifically, the tactile modality has been, in general, ignored in the SL literature.

Research would clearly benefit from more longitudinal data, which can inform on the predictive role of early SL skills for later language outcomes. In particular, the directionality of the association between language competence and SL skills needs to be established in controlled designs, especially given that the current review provides some evidence of a bi-directional relationship, which may be due to inconsistencies in the study designs and/or selection of analyses. Most studies in the current review have employed explicit measures, such as e.g., forced choice tasks. The latter have been criticized for not adequately capturing individual differences [11,71,72]. Future studies will thus benefit from using implicit online measures, especially given the temporal nature of SL. Neurophysiological measures might be of particular interest due to the opportunity to analyze the overlap of brain activity in areas associated with SL and language acquisition, especially because SL has been shown to make use of areas heavily implicated in language, such as the left inferior frontal gyrus [74], as well as areas which contribute to memory and associative learning, such as the medial temporal lobe [75]. A promising perspective is to acknowledge the presence of distinct components in the process underlying SL., It has been suggested that statistical learning can be broken down into two dissociable components: (1) perceptual binding of individual stimulus units into integrated composites and (2) storing those integrated representations for later use [76]. Such a view is consistent with the evidence from word learning in children, whereby the initial stages involve speech segmentation and consolidation of the phonological form, which can subsequently be used for the purposes of mapping onto content [20,77,78]. These two respective components of SL can, and should, be studied independently with specifically designed tasks. While typical statistical learning tests which rely on post-learning tasks, conflate these two components, monitoring the critical component of learning via rhythmic EEG entrainment may reveal the gradual acquisition of knowledge, whereby novel stimulus sequences are transformed into familiar composites. In a series of studies [76], have demonstrated that this online perceptual transformation is a critical component of learning and can be reliably studied using neural oscillation data. Furthermore, this research indicates that both children and adults demonstrate robust neural entrainment to words and syllables, paralleled by comparable behavioural results on explicit SL tasks [79]. However, for the children, unlike the adults, the increase of entrainment to words was not accompanied by a decrease in entrainment to syllables, as shown by the inter-trial phase coherence (ITC) measure for syllables. This latter result is interesting and may suggest a mechanism to synchronize both at the level of the word and the syllable, in all likelihood related to heightened word segmentation skills in childhood. Importantly, and consistent with recent criticism of explicit Forced Choice Tasks [11,71,72], this study found only weak relationships between the neural entrainment tasks and the explicit SL tasks. These results are also consistent with findings of neural entrainment to word-like units in the absence of any behavioural evidence of learning [80,81]. This suggests that performance on explicit tasks, in addition to tapping SL, recruits other mechanisms, such as those underlying the Executive Function System [1,66].

## 6 Limitations

Scoping reviews provide a map of the literature, summarize evidence, and inform about knowledge gaps [34], and the current review tried to be as systematic as possible, by following the JBI methodology for scoping reviews. However, it lacks the statistical synthesis of a meta-analysis, which could inform on the statistical strength of the association across studies. A meta-analysis, however, would not have been informative, because of the methodological heterogeneity of the studies, particularly regarding the characteristics of the SL tasks. In fact, although we organized the tasks in broad categories (for the sake of presenting them clearly), we seldom found a study which employed exactly the same methodology as its original paradigm. This gives rise to a great number of tasks, varying in number of trials, stimuli characteristics, experiment duration etc.

In this review, and according to established scoping review practice, we opted not to search for unpublished studies. A large percentage of the studies included here reported non-significant results (32%), and it remains unclear to what extent these results might still be affected by publication bias. For example, the fact that even published studies report non-significant results might suggest that others may have obtained non-significant results too, which were never published. We also excluded non peer-reviewed publications and secondary research. Although these can be included in a scoping review, we aimed at some homogeneity and therefore looked for literature with comparable standards.

## 7 Conclusions

Our findings support the SL account of language acquisition by revealing SL-based individual differences in language competence measures. Typically developing infants and children with stronger auditory and audio-visual SL skills perform better on lexical competence tasks. Due to the scarcity of studies, it is still unknown whether auditory and audio-visual SL also play a role in other linguistic domains. Our results also open up the question of the role of visual SL for language development, and the possibility of selective associations between specific SL modalities and language domains, pending future research. These results indicate that the relevance of SL skills for language development is dependent on sensory modality.

Research on SL individual differences in language acquisition is biased towards lexical measures and auditory/visual SL measures. More studies are needed in non-lexical domains and in other sensory modalities. Studies should apply longitudinal designs to more consistently inform about how SL skills predict language proficiency.

## Author contribution statement

All authors listed have significantly contributed to the development and the writing of this article.

## Funding information

This project has received funding from the European Union's Horizon 2020 research and innovation programme under the Marie Skłodowska-Curie grant agreement No. 857897.

## Data availability statement

Data associated with this study has been deposited at Open Science Framework (registration https://doi.org/10.17605/OSF.IO/CEQH5).

## Declaration of competing interest

The authors declare that they have no known competing financial interests or personal relationships that could have appeared to influence the work reported in this paper.

## Appendix A Supplementary data

Appendix A.docx Appendix A.docx

Appendix B.docx Appendix B.docx
