# Supplementary material for: The association between statistical learning and language development during childhood: A scoping review
Source: Heliyon. 2023 Jul 26;9(8):e18693. doi: 10.1016/j.heliyon.2023.e18693 (PMC10405008; doi:10.1016/j.heliyon.2023.e18693)
Supplement: Appendix A.docx [file mmc1.docx]

**Appendix A – Individual search strategies for each of the databases searched.**

| **Database** | **Search query** | **Filters** | **Records retrieved** | **Date of the search** |
| --- | --- | --- | --- | --- |
| PubMed | (("statistical learning") OR ("artificial language") OR ("artificial grammar"))  AND  (("language") OR ("phonology") OR ("vocabulary") OR ("lexicon") OR ("grammar") OR ("syntax") OR ("morphology"))  AND  (("infants") OR ("toddlers") OR ("children") OR ("preschoolers") OR ("school children")) | English language | 294 | 15/02/21 |
| Web of Science | ALL=("statistical learning" OR "artificial language" OR "artificial grammar")  AND  ALL=("language" OR "phonology" OR "vocabulary" OR "lexicon" OR "grammar" OR "syntax" OR "morphology")  AND  ALL=("infants" OR "toddlers" OR "children" OR "preschoolers" OR "school children") | English language | 672 | 15/02/21 |
| PSYCINFO (Ovid) | ("statistical learning" OR "artificial language" OR "artificial grammar")  AND  ("language" OR "phonology" OR "vocabulary" OR "lexicon" OR "grammar" OR "syntax" OR "morphology")  AND  ("infants" OR "toddlers" OR "children" OR "preschoolers" OR "school children") | Peer reviewed English language | 4214 | 18/02/21 |
| EBSCOHost | ("statistical learning" OR "artificial language" OR "artificial grammar")  AND  ("language" OR "phonology" OR "vocabulary" OR "lexicon" OR "grammar" OR "syntax" OR "morphology")  AND  ("infants" OR "toddlers" OR "children" OR "preschoolers" OR "school children") | Peer reviewed English language | 670 | 18/02/21 |
