# Supplementary material for: The association between statistical learning and language development during childhood: A scoping review
Source: Heliyon. 2023 Jul 26;9(8):e18693. doi: 10.1016/j.heliyon.2023.e18693 (PMC10405008; doi:10.1016/j.heliyon.2023.e18693)
Supplement: Appendix B.docx [file mmc2.docx]

**Appendix B – Revised data extraction instrument.**

| **Reference** |
| --- |
| **Design & sample** |
| Research design (cross-sectional, longitudinal) |
| Age of the participants |
| Native language of the participants |
| Sample size |
| **Language development test** |
| Domain |
| Test/task name |
| **Statistical learning task** |
| Sensory modality |
| Sensory modality complexity (unimodal/multimodal) |
| Task name |
| Stimuli |
| Statistical pattern |
| Measure of learning (e.g., looking times) |
| Explicit/implicit learning measure |
| Behavioural/neurophysiological learning measure |
| **Statistical analysis of the association** |
| Test |
| Results |
